# Identifying Meteorological Drivers for the Seasonal Variations of Influenza Infections in a Subtropical City — Hong Kong

**DOI:** 10.3390/ijerph120201560

**Published:** 2015-01-28

**Authors:** Ka Chun Chong, William Goggins, Benny Chung Ying Zee, Maggie Haitian Wang

**Affiliations:** 1 Division of Biostatistics, The Jockey Club School of Public Health and Primary Care, The Chinese University of Hong Kong, Hong Kong, China; E-Mails: wgoggins@cuhk.edu.hk (W.G.); bzee@cct.cuhk.edu.hk (B.C.Y.Z.); 2 Clinical Trials and Biostatistics Lab, Shenzhen Research Institute, The Chinese University of Hong Kong, Shenzhen, China

**Keywords:** temperature, influenza, seasonality, transmission rate, epidemic, SIR model

## Abstract

Compared with temperate areas, the understanding of seasonal variations of influenza infections is lacking in subtropical and tropical regions. Insufficient information about viral activity increases the difficulty of forecasting the disease burden and thus hampers official preparation efforts. Here we identified potential meteorological factors that drove the seasonal variations in influenza infections in a subtropical city, Hong Kong. We fitted the meteorological data and influenza mortality data from 2002 to 2009 in a Susceptible-Infected-Recovered model. From the results, air temperature was a common significant driver of seasonal patterns and cold temperature was associated with an increase in transmission intensity for most of the influenza epidemics. Except 2004, the fitted models with significant meteorological factors could account for more than 10% of the variance in additional to the null model. Rainfall was also found to be a significant driver of seasonal influenza, although results were less robust. The identified meteorological indicators could alert officials to take appropriate control measures for influenza epidemics, such as enhancing vaccination activities before cold seasons. Further studies are required to fully justify the associations.

## 1. Introduction

Hong Kong, a city located in the South China Sea, has a humid subtropical climate with winter (December–February) temperatures that usually range from 10 to 20 °C, warm springs and autumns, and hot summers (June–September) with daytime temperatures in the low to mid 30 s and nighttime temperatures in the high 20 s. In temperate regions, influenza has a clear seasonal pattern with an exponential increase in infections in the winter, which is followed by a fade-out period of a few months. In subtropical regions, there is no sufficient understanding of the seasonal pattern of influenza and its relationship with meteorological factors. The number of epidemic peaks can differ across various subtropical regions, with the peaks usually occurring at different periods within a year [1,2,3]. According to the World Health Organization (WHO), influenza epidemics result in 250 to 500 thousand deaths worldwide annually [4]. In Hong Kong, the influenza hospitalization rate and the pneumonia and influenza (P&I) associated mortality were estimated to be 29 and 4.1 per 100,000 person-years respectively [5,6]. Hong Kong was also regarded as an epicenter of pandemic influenza in Southeast Asia. Insufficient information about viral activity creates difficulties in forecasting the disease burden and thus hampers official preparation efforts.

Despite numerous researches that have discovered meteorological factors associated with various activities of influenza, little is known about the drivers of transmission or its seasonal variations for different climates. A small variation in influenza transmission could result in amplification and damping of infection oscillations over time and thus sustain a seasonal pattern [7]. Possible drivers of influenza transmission include meteorological variations [8], susceptible numbers [9,10], and social mixing [3]. Recently, Shaman* et al.* employed a mathematical model to demonstrate that the seasonal pattern of influenza in the United States could be drawn based on the process of simulations driven by the absolute humidity [8]. This finding motivated a further investigation of potential meteorological drivers for subtropical climates.

In this study, meteorological determinants that could drive the seasonal variations of influenza in Hong Kong were investigated by a mathematical model. We hypothesized that the transmission rates in a population-level model, as well as the infection oscillations of seasonal influenzas, are affected by meteorological factors. Identification of the drivers will help to improve the understanding of influenza transmission and to alert officials to implement preemptive control measures for seasonal influenza.

## 2. Materials and Methods

### 2.1. Data

**Figure 1:**
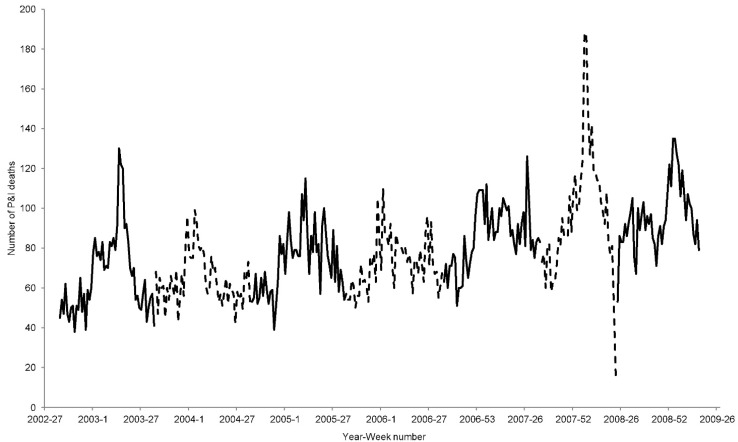
Number of P&I deaths from 2002 to 2009 in Hong Kong. The alternating solid and dashed line separates the yearly data.

Data on deaths from P&I from 2002 to 2009 in Hong Kong were obtained from the Hong Kong Census and Statistics Department (Figure 1). The mid-year population (*N_year_*) from 2002 to 2008 was collected from the Hong Kong Census and Statistics Department [11]. We separated each wave of P&I deaths by year from week 35 to week 34 of the following year. As the wave of 2007 was stopped earlier, it would start from week 35 until week 23 of 2008. The 2008 wave is from week 24 to week 16 of 2009, in order to prevent the overlap of cases after the outbreak of the 2009 H1N1 pandemic.

**Figure 2:**
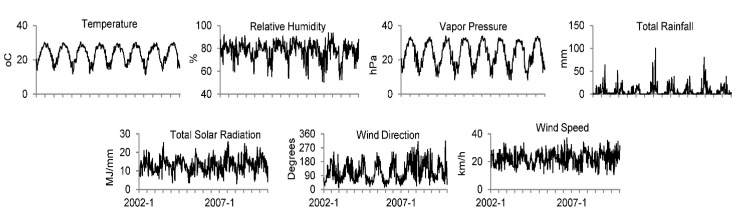
Weekly average of meteorological parameters from 2002 to 2009 in Hong Kong.

The weekly average of meteorological parameters: air temperature, relative humidity, total rainfall, total solar radiation, wind direction, and wind speed from 2002 to 2009 were collected from the Hong Kong Observatory. The time series of the data is shown in Figure 2. Actual vapor pressure (*e*) was calculated as a metric for absolute humidity by the Teten’s formula [12,13]:

(1) $$e=\frac{rh}{100}\times e_{s}(Ta)$$

where *e_s_*(*Ta*) is the saturation vapor pressure (hPa), *rh* is the relative humidity (%), and *Ta* is the air temperature (°C). The *e_s_* was calculated as follows:

(2) $$e_{s}(Ta)=6.105\times \exp\left(\frac{17.27\times Ta}{237.7+Ta}\right)$$

The saturation vapor pressure in the Teten’s formula can also be obtained by the integration of Clausius-Clapeyron equation and is acceptable for most meteorological purposes [12,14]. As the wind data was in the polar coordinate scale, we develop wind velocity variables in the Cartesian scale that encompasses wind direction and wind speed, thus preventing the problem of northerly bearings being split at true north. Two parameters of wind velocity (East-to-West and North-to-South) were used as metrics for wind data in the analysis.

### 2.2. Mathematical Model

We extended the Susceptible-Infected-Recovered (SIR) model from Chowell* et al.* [15] to describe the dynamic system of seasonal influenza. In this model, a population is comprised of four compartments: susceptible (*S(t)*); infectious (*I(t)*); recovered (*R(t)*); and dead (*D(t)*), at each time point *t*. The SIR model consists of four differential equations that describes the rates of subject movements for each of the time steps. We assumed homogeneous mixing, meaning that each individual has the same chance of contacting another individual within the population. In the compartmental model, once susceptible individuals in compartment *S(t)* get infected, they will move to compartment *I(t)* and stay there for the infectious period. When the infectious period is over, the individuals in compartment *I(t)* will recover and move to compartment *R(t)* or will die and move to compartment *D(t)*. In this model, the time-varying transmission rate per individual is β*_t_* and the force of infection for time *t* is β*_t_I*. We denote *S(t)*, *I(t)*, *R(t)*, and* D(t)* as *S*, *I*, *R*, and *D* as the subpopulations in each compartment for time *t*. The deterministic system of equations are as follows:

(3) $$\begin{array}{l} \frac{dS}{dt}=-{\text{β}}_{t}SI\\ \frac{dI}{dt}={\text{β}}_{t}SI-(\text{γ}+\text{δ})I\\ \frac{dR}{dt}=\text{γ}I\\ \frac{dD}{dt}=\text{δ}I \end{array}$$

We assumed that the length of the generation interval (GI) follows an exponential distribution with mean = 1/(γ *+* δ*)*. Suppose CFP is the average case fatality proportion, the mortality rate is δ = (CFP/(1 − CFP)) and δ*I* is the influenza deaths generated by the differential equations. To make the model coefficients more comparable to each other, meteorological variables are transformed by subtracting the mean and divided by the standard deviation (SD) over their sampling period. Let *X_t_^i^* be a particular *i*-th independent variable (e.g., air temperature at time *t*), the transformed form would be:

(4) $${Z_{t}}^{i}=\frac{{X_{t}}^{i}-{\bar{X}}^{i}}{{\text{σ}}_{X^{i}}}$$

where ${\bar{X}}^{i}$ is the sample mean and ${\text{σ}}_{X^{i}}$ is the SD for the sampling period. The meteorological effects are related to β*_t_* using the following linear component:

(5) $${\text{β}}_{t}=b_{0}+b_{1}{Z_{t}}^{1}+b_{2}{Z_{t}}^{2}+...+b_{n}{Z_{t}}^{n}$$

where *n* is the number of independent variables. The model will determine the significant drivers to the influenza transmission rate.

### 2.3. Parameter Estimation

In the differential equations, we assumed a 5-day length of GI [16] and a 0.2% CFP [17]. To account for the variation of partial immunity to the seasonal influenza, we followed previously published procedures [15,18]. The initial number of susceptibles in Equation (3) was calculated by *S(0) = N_year _− I(0) − R(0) − D(0)*, where *D(0)* was set to be the number of P&I deaths in the first epidemic week. Thus, the initial number of recovered individuals can be calculated as *R(0)* =* D(0)/*CFP *− D(0)*. Rather than fixing a value, mid-year population (*N_year_*) was used for each wave, so as to reduce the impact from natural mortality and birth.

The weekly P&I death data was fitted to model generated deaths (*i.e.*, δ*I*) and the meteorological time series data consisted of the variables (*X_t_^i^*) for each epidemic wave. Parameters *I(0)*, *b_0_*, *b_1_*,…,*b_n_* could be estimated by least-squares fitting to the data. As weekly data was used, *t* was measured in weeks. Statistically significant meteorological parameters (*p*-value of *b_i_* < 0.05) were declared as potential drivers to seasonal variations of influenza. A stepwise variable selection approach was adopted and the best fitted model was chosen as the one with all statistically significant variables and the lowest Akaike Information Criterion (AIC) [19]:

(6) $$AIC=m\log\left(\frac{SSE}{m}\right)+2p$$

where *m* is the number of data points, *p* is the total number of parameters, and *SSE* is the sum of square errors. Instead of sampling, all possible parameter combinations were assessed by a grid search. As the absolute humidity was derived from the temperature and relative humidity, they could not be included in the same variable pool during the stepwise variable selection, due to the co-linearity problem. The variable set with temperature, relative humidity, plus other variables and the set with absolute humidity plus other variables were separately adopted in the variable selection in order to draw two final models. The best fitted model was then chosen based on AIC value (lower being better). Adjusted R-square (Adj-R^2^) is the measure of proportion of variance explained by the model after the parsimony adjustment. The difference of Adj-R^2^ between the null model and fitted model (ΔAdj-R^2^) was interpreted as the proportion of variance explained by the meteorological factors.

### 2.4. Sensitivity Analysis

We conducted a sensitivity analysis addressing two aspects:

(1) *Model parameters*: Sensitivity analysis was performed by varying the length of the GI for 3 days and 7 days [16] and the CFP for 0.1% and 0.4% [17].
(2) *Model structure*: In addition to the linear form of Equation (5), a multiplicative exponential form was also adopted in model fitting to test whether this would produce different results:
  (7) $${\text{β}}_{t}=b_{0}\exp(b_{1}{Z_{t}}^{1}+b_{2}{Z_{t}}^{2}+...+b_{n}{Z_{t}}^{n})$$

## 3. Results

### 3.1. Model Goodness of Fit

**Figure 3:**
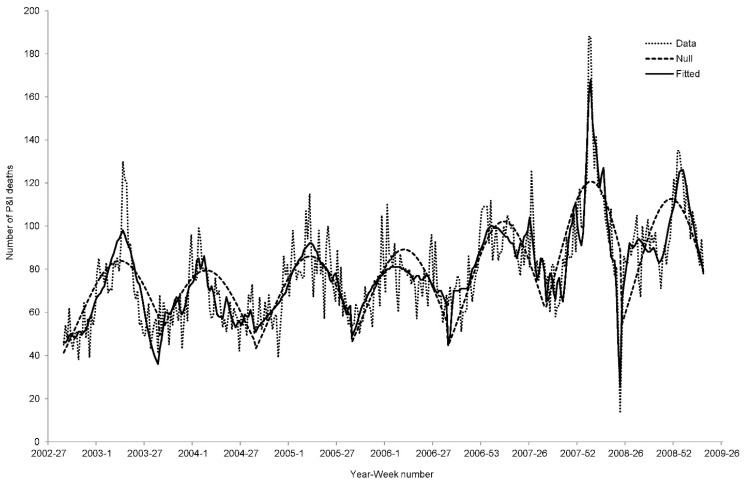
Fitted curves from the null model and the best fitted model.

Table 1 summarizes the results of the best fitted models with lowest AIC and all statistical significant meteorological parameters. Compared with the null models (β*_t_* = constant), models with meteorological parameters had better goodness of fit in terms of their AIC (Figure 3). No null model was found to be the best fitting model after the stepwise variable selection. With the exceptions of the 2003 and 2005 P&I waves, Adj-R^2^ was always greater than 40%, indicating that models with selected meteorological parameters explained more than 40% of the variance in P&I mortality after the adjustment of number of parameters. Except for 2004, the models with significant meteorological factors could account for more than 10% of variance in addition to the null model (*i.e.*, ΔAdj-R^2^ > 10%). For the 2008 epidemic, the meteorological parameters accounted for more than 50% of the variability in the P&I data. Nevertheless, the P&I data of the 2004 epidemic could not be well explained by the best fitted models.

**Table 1:** Estimates (standard error) of significant meteorological determinants on changes of influenza transmission rate.

Variables ^a^	Year						
	2002	2003	2004	2005	2006	2007	2008
Temperature	−4.1 (0.8)	−9.8 (2.9)	−2.6 (1.2)		−2.9 (1.2)	−5.2 (1.0)	−3.7 (0.6)
Rel. humidity	3.6 (1.4)						−3.5 (0.7)
Abs. humidity							
Rainfall		10.7 (3.1)	4.5 (2.0)	2.3 (0.6)	9.8 (2.3)	−12.7 (3.9)	5.5 (1.9)
Solar radiation		10.9 (3.4)					
Wind velocity (EW)		7.6 (3.5)				−12.5 (2.8)	
Wind velocity (NS)						10.9 (3.1)	
AIC	262.6	246.8	258.9	251.1	262.4	216.8	208.7
Adj-R^2^ (%)	66.5	37.1	48.8	32.9	47.4	84.0	67.2
ΔAdj-R^2^ (%)	18.9	22.1	2.9	16.1	32.9	38.7	56.9

**^a^** Estimates of meteorological variable were in 10^−9^ unit; Rel. humidity: Relative humidity; Abs. humidity: Absolute humidity; Wind velocity (EW): Wind velocity for East-to-West; Wind velocity (NS): Wind velocity for North-to-South; AIC: Akaike information criterion; Adj-R^2^: Adjusted R-square in %; ΔAdj-R^2^: Difference of adjusted R-square between null model and fitted model.

### 3.2. Significant Meteorological Determinants

As shown in Table 1, air temperature and rainfall were the most common significant variables driving the seasonal variations of the P&I waves from 2002 to 2008. The air temperature was negatively associated with the time-varying transmission rate β*_t_* in six of the seven epidemics; for one SD decrease in temperature, the transmission rate would increases by 4.1, 9.8, 2.6, 2.9, 5.2, 3.7 (×10^−9^) for the years 2002, 2003, 2004, 2006, 2007 and 2008 respectively. Moreover, rainfall was positively associated with the transmission intensity in five of the seven epidemic waves. When there was a SD increase in rainfall, the transmission rate would increases by 10.7, 4.5, 2.3, 9.8, 5.5 (×10^−9^) for the years 2003–2006, and 2008 respectively. A negative association was found for 2007. Surprisingly, relative humidity and absolute humidity did not show much contribution to the variance of β*_t_* among all the P&I epidemics.

### 3.3. Sensitivity Analysis

**Figure 4:**
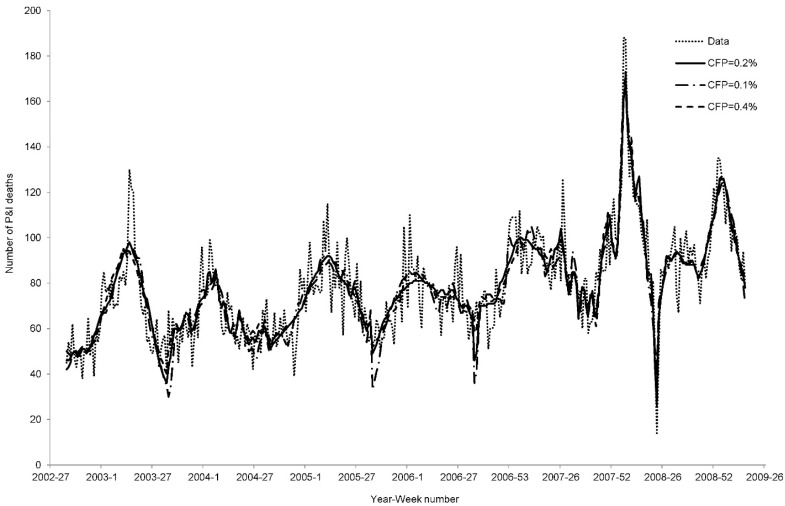
Best fitted curves for CFP = 0.2%, 0.1%, and 0.4%.

**Figure 5:**
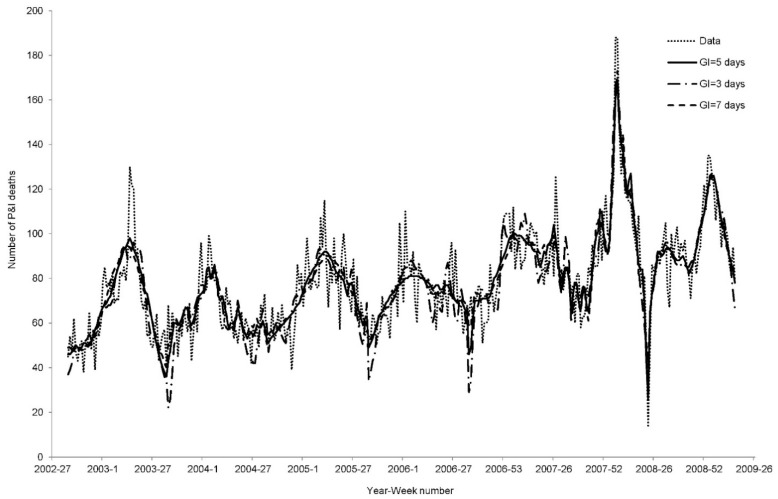
Best fitted curves for GI = 5 days, 3 days, and 7 days.

A sensitivity analysis was conducted to test the impact of our results from different parameter settings. In brief, varying the CFP (0.1% and 0.4%) and GI (3 and 7 days) only produced a slight effect on the goodness of fits. As shown in Figure 4 and Figure 5, the best fitting curves were highly similar. In terms of AIC and Adj-R^2^, no significant differences were produced as a result of using different CFP and GI settings (Table 2 and Table 3). The fitness of the models with either CFP = 0.1% or GI = 3 days were worse than the other models in several epidemic waves.

**Table 2:** Estimates (standard error) of significant meteorological determinants on changes of influenza transmission rate when CFP = 0.1% or CFP = 0.4%.

**CFP = 0.1%**	**Year**						
**Variables ^a^**	**2002**	**2003**	**2004**	**2005**	**2006**	**2007**	**2008**
Temperature	−6.8 (2.0)	−9.3 (3.3)	−3.5 (1.5)		−7.7 (1.6)	7.6 (0.9)	−6.6 (0.3)
Rel. humidity	7.8 (1.5)		2.8 (1.0)			2.6 (1.2)	
Abs. humidity							
Rainfall		14.4 (2.9)	9.9 (3.2)	7.3 (0.7)	16.2 (2.9)		
Solar radiation	8.4 (3.2)	11.5 (3.9)			9.5 (2.4)		
Wind velocity (EW)						−10.6 (2.9)	
Wind velocity (NS)			5.7 (2.7)		9.3 (1.8)	9.1 (3.2)	
AIC	268.7	261.2	264.4	261.8	271.3	218.5	211.3
Adj-R^2^ (%)	62.9	15.6	45.1	17.6	40.0	83.4	63.8
**CFP = 0.4%**	**Year**						
**Variables**	**2002**	**2003**	**2004**	**2005**	**2006**	**2007**	**2008**
Temperature	−4.4 (0.6)	−10.0 (2.9)	−1.6 (0.5)			−5.7 (1.0)	−2.3 (0.6)
Rel. humidity				−2.4 (0.9)		−6.7 (1.2)	−4.8 (0.7)
Abs. humidity					−3.3 (1.5)		
Rainfall		8.1 (3.1)		2.2 (1.0)	6.7 (2.8)		7.3 (1.9)
Solar radiation		11.0 (3.4)					
Wind velocity (EW)		9.2 (3.4)				−14.0 (2.9)	
Wind velocity (NS)						11.4 (3.3)	
AIC	261.6	245.3	257.2	248.5	260.6	221.4	207.7
Adj-R^2^ (%)	66.5	38.8	49.6	37.3	49.2	82.2	68.0

**^a^** Estimates of meteorological variable were in 10^−9^ unit; Rel. humidity: Relative humidity; Abs. humidity: Absolute humidity; Wind velocity (EW): Wind velocity for East-to-West; Wind velocity (NS): Wind velocity for North-to-South; AIC: Akaike information criterion; Adj-R^2^: Adjusted R-square in %.

**Table 3:** Estimates (standard error) of significant meteorological determinants on changes of influenza transmission rate when GI = 3 days or GI = 7 days.

**GI = 3 days**	**Year**						
**Variables ^a^**	**2002**	**2003**	**2004**	**2005**	**2006**	**2007**	**2008**
Temperature	−7.8 (2.1)	−9.4 (3.7)	−3.6 (1.5)	−4.1 (1.5)	−10.4 (1.8)	−8.2 (0.9)	−7.1 (0.8)
Rel. humidity	10.4 (1.6)		4.8 (1.1)			7.2 (1.2)	
Abs. humidity							
Rainfall		19.3 (3.3)	12.6 (3.2)	15.5 (1.9)	21.3 (3.3)		
Solar radiation	13.0 (3.4)	11.3 (4.3)			14.0 (2.7)		−4.0 (1.6)
Wind velocity (EW)						−8.5 (3.0)	
Wind velocity (NS)			6.7 (2.8)		13.8 (2.0)	7.7 (3.2)	
AIC	274.4	273.1	269.6	271.3	282.9	221.6	216.4
Adj-R^2^ (%)	58.6	12.3	39.2	15.6	25.2	82.1	60.3
**GI=7 days**	**Year**						
**Variables**	**2002**	**2003**	**2004**	**2005**	**2006**	**2007**	**2008**
Temperature	−4.4 (0.6)	−10.1 (2.9)	−1.6 (0.5)			−5.8 (1.0)	−2.3 (0.6)
Rel. humidity				−2.4 (0.9)		−6.7 (1.2)	−4.8 (0.7)
Abs. humidity					−3.3 (1.5)		
Rainfall		8.2 (3.1)		2.2 (1.0)	6.9 (2.8)		7.3 (1.9)
Solar radiation		11.1 (3.4)					
Wind velocity (EW)		9.3 (3.4)				−14.1 (3.0)	
Wind velocity (NS)						11.5 (3.3)	
AIC	261.6	245.3	257.1	248.5	260.5	221.4	207.7
Adj-R^2^ (%)	66.5	38.9	49.6	37.2	49.3	82.2	68.0

**^a^** Estimates of meteorological variable were in 10^−9^ unit; Rel. humidity: Relative humidity; Abs. humidity: Absolute humidity; Wind velocity (EW): Wind velocity for East-to-West; Wind velocity (NS): Wind velocity for North-to-South; AIC: Akaike information criterion; Adj-R^2^: Adjusted R-square in %.

The effect of temperature was only slightly sensitive to the variation in CFP and GI. In most situations, air temperature continued to be identified as a common driver of seasonal variations. When GI = 3 days, air temperature significantly drove the variations in all epidemics. Whereas the effect of rainfall was moderately sensitive to variations in CFP and GI. Rainfall was identified as the significant driver of four of the studied influenza epidemics. The decrease of rainfall’s significance may be due to the model variance shared with relative humidity. The meteorological variable selection was not sufficiently sensitive, even if the model structure was changed to the exponential form (Table S1).

## 4. Discussion

Recent studies have demonstrated that environment factors account for a proportion of the seasonality, as well as infection oscillations, of influenzas in temperate regions [8,9]. Here we used a mathematical model to explore the potential meteorological drivers for seasonal oscillations of influenza in a subtropical city, Hong Kong. Through modulating the transmission rates by the meteorological factors in an infectious disease model, the seasonal variations of influenza infections could be well-depicted. According to our results, although no meteorological parameters dominated the seasonal variations for all epidemics, air temperature significantly modulated the fluctuations of transmission rates for most of the epidemics between 2002 and 2009. Rainfall was also found to be a significant driver for most of the epidemics, although its direction of association was not unidirectional and it was moderately sensitive to changes in the model parameters.

In many laboratory and epidemiological studies, air temperature is often found to be associated with influenza transmissions [1,20,21,22,23,24]. An epidemiological study from Chan* et al.* [1] found that temperature and relatively humidity were associated with the activity of seasonal influenza in Hong Kong; a cold and humid climate was related to higher activities of both influenza A and B. Lowen* et al.* [21] conducted an experimental study using a guinea pig model to demonstrate that cold temperature favored to the spread of the influenza virus. Our study extended these findings by showing that cold temperature was associated with the mechanism driving seasonal oscillations at a population level. This is perhaps due to prolonged survival of viral particles under colder conditions. Nevertheless, the effect of temperature could be confounded by other factors [25]. For example, a decrease in temperature could enhance crowding at indoor activities, and would thus increase the contact, aerosol and droplet transmission intensity.

In our study, we could not identify any strong evidences that absolute humidity drove the seasonal variability in Hong Kong, even though experimental and modeling studies have shown that absolute humidity was related to viral survivorship and was capable of driving the seasonality of influenza in temperate regions [9,10,25]. One possible explanation for this is that the absolute humidity in Hong Kong was high all year around, compared to temperate areas. Like other tropical and subtropical regions, use of air conditioning is common in Hong Kong when the temperature is high. One could argue that, using air conditioning would lower the indoor absolute humidity and thus modulate the survivorship of the influenza virus. The effect from air exchange would indeed offset the impact of disease transmission.

In addition to cold temperature, experimental studies have indicated that a low relative humidity could enhance the influenza transmission [21]. According to our results, relative humidity was not identified as a significant driver for the seasonal variation of influenza infection. This might be accounted for by the relative unpopularity of indoor heating in Hong Kong compared to temperate regions. Moreover, the predominant mode of influenza virus spread was proposed to be different between temperate and tropical regions [26]. Relative humidity would be more insensitive for transmissions by the contact route than by the aerosol route.

Previous studies shown that rainfall could be used as a predictor to forecast influenza infection rates for sub-tropical regions, but not in all temperate regions [27]. These authors also indicated that rainfall was correlated with seasonal influenza transmission in Hong Kong [20], and this finding was in line with other tropical areas [28]. Nevertheless, there remains no clear and definitive explanation for the mechanism of rainfall driving the influenza seasonality. Although low temperature and dry air have been proven to be favorable for survival of viral particles [22], no study has investigated the relationships between rainy conditions and bulk aerosol transport. One plausible mechanism is that rainfall could affect human social behaviors, such as indoor activities, and therefore influence the number of contacts and the risk of exposure to contaminated environments or infected individuals. In our study, we found that rainfall significantly drove some epidemics but that its direction of association was not unidirectional, likely due to the problem of multicollinearity, which has been investigated in our association analysis (Table S2).

Solar radiation could cause seasonal variations in vitamin D photosynthesis that may affect immune responses as well as playing a role in the influenza seasonality [29,30]. The preventive efficacy of vitamin D supplementation against influenza infections has also been demonstrated in trial studies [31]. Our study did not identify solar radiation as a driver for the seasonality of influenza infection. This result was not surprising because the effect of solar radiation on the population of the subtropics is not as well documented as in temperate regions. In addition, influenza A would be more likely affected by vitamin D status than influenza B [31]. This factor might confound our findings when P&I data was adopted in the study. Nevertheless, the role of solar radiation in seasonality remains controversial because it has been difficult to explain the influenza dynamic in outdoor environments; most transmissions occur in indoor environments through airborne transmission or contact [32].

Rather than pooling all of our data, the purpose of conducting the analysis by seasons was to investigate the meteorological effects independent of the between-season variations. The between-season effect was made up by “nuisance variables”, which could result from variations of reporting rate and other potential factors that affected the susceptibility numbers [10], such as the vaccination effectiveness. Although the partial immunity of the seasonal influenza was adjusted in our analysis, some factors are difficult to measure and interpret. Analysis by seasons could confound the relationship between meteorological factors and transmission rate, and thus generate inconsistency for the estimated coefficients (e.g., a negative association of rainfall in 2007). An additional analysis was conducted using the pooled data and the results were summarized in supplementary Table S3. By this approach the main finding was unchanged (*i.e.*, a low temperature drove the influenza transmission). It should be noted that between-season effect accounted for 20% of the total variance in addition to the meteorological determinants.

## 5. Study Limitations and Uncertainties

In our study, there is undoubtedly some degree of correlation between meteorological variables. Hence, we additionally conducted a correlation analysis in which the Pearson correlation coefficients and variance inflation factors (VIF) were drawn from the pooled data, with the results summarized in supplementary Table S2. Although positive correlations were found between temperature and solar radiation, and between rainfall and humidity, no serious effect of multicollinearity was found for any of the predictors based on a simple rule-of-thumb (*i.e.*, all VIFs were less than 3).

One limitation of our study is that we only investigated the environmental drivers for disease transmission and could therefore not completely rule out confounding factors. According to some studies [8,9,10,22], some seasonal changes of host behavior (e.g., international travel [33,34] and school holidays [35]) might also affect the transmission dynamics. It has been shown that the closure of kindergartens and primary schools was able to reduce the disease transmission rate by around 25% for the 2009 influenza A/H1N1 pandemic. Nevertheless, its effect upon the seasonal variation of influenza is controversial. Some studies [10,36] pointed out that no substantial effect on the transmission reduction could be detected when schools were closed. In addition, our results were undoubtedly affected by the demographics of the population (e.g., age and gender). Subjects with different clinical status, such as chronic obstructive pulmonary disease, may also have confounded the likelihood of P&I deaths. The sufficiency of details to address these issues requires a huge effort in data collection, which remains difficult to achieve at this stage. Further research is warranted to investigate the effects of seasonal social/behavior patterns.

A limitation to our study is that the use of P&I mortality to represent the influenza activity may not be completely adequate and could potentially bias the findings. Although some studies have preferred using P&I mortality [15], we also analyzed the P&I excess mortality to test the robustness of the study finding. We adopted the traditional Serfling approach to estimate the excess mortality [37,38]. The Serfling method is a linear regression model using harmonic terms to calculate the expected mortality in the absence of influenza virus activity. The number of excess deaths attributable to influenza was estimated as the difference between the observed and the upper 95% limit of the prediction interval of baseline deaths. The details were noted in supplementary Table S4. From the results, the principal finding was unchanged (*i.e.*, air temperature remained a significant driver of the seasonal patterns). It should be noted that no climatic variables can be fitted into the year 2003 due to few P&I excess mortality. This might be due to the mitigation measures for the severe acute respiratory syndrome (SARS) epidemic that potentially also reduced the number of influenza cases [39].

Undoubtedly, it has been widely recognized that the disease severity of influenza, in terms of excess P&I deaths and hospitalization, tended to be higher in the influenza A dominant seasons than in those with influenza B as the dominant virus strains [5]. Moreover, some influenza B epidemics resulted in increased hospitalizations but not increased mortality [40]. As a result, our findings might be less precise for mild influenza seasons and may not reflect the general influenza experience in Hong Kong. This is a common limitation in studies that employed mortality surveillance. In addition to death data, influenza-like illness (ILI) surveillance has been commonly adopted as a proxy for influenza activity. Nevertheless, the definition of ILI failed to document significant influenza-associated morbidity and mortality [5]. As such, ILI is a poor indicator of influenza activity when adopted in areas with a less defined pattern of seasonality [41]. Laboratory surveillance data would be a better indicator for influenza activity but large efforts would be required to gather and collate such data.

The data used in this study was only applied the retrospective fitting. Further studies would have to be conducted to validate these results. For example, more data is required to extend the model application to projecting the SIR curve for model validations [8]. Plausible causality and potential interactions should also be justified, such as direct and indirect effects of air temperatures [22]. Nevertheless, our study represents an initial step towards identifying potential meteorological determinants for driving the seasonal variations of influenza in a subtropical region.

## 6. Conclusions

This study identified the potential meteorological drivers for the seasonal variations of influenza in a subtropical city, Hong Kong. Results show that the cold air temperature was a significant driver for increasing the transmission intensity of seasonal influenza from 2002 to 2009. Rainfall was also found to be a significant driver for some seasons, although this result was less robust. An accurate would enable officials to take appropriate control measures for influenza epidemics, such as maintaining sufficient indoor temperature and enhancing vaccination activities prior to the cold seasons. Further laboratory and epidemiological studies are required to validate and justify the associations proposed here.

## Supplementary Files

Supplementary File 1
