# Supplementary material for: Identifying Meteorological Drivers for the Seasonal Variations of Influenza Infections in a Subtropical City — Hong Kong
Source: Int J Environ Res Public Health. 2015 Jan 28;12(2):1560–76. doi: 10.3390/ijerph120201560 (PMC4344680; doi:10.3390/ijerph120201560)
Supplement: Supplementary File 1 [file ijerph-12-01560-s001.pdf]

Supplementary Information

Identifying Meteorological Drivers for the Seasonal Variations of Influenza Infections in a Subtropical City — Hong Kong

Table S1. Estimates (standard error) of significant meteorological determinants on changes of transmission rates when fitting into a multiplicative model.

Variables [a]	Year						
	2002	2003	2004	2005	2006	2007	2008
Temperature	−1.9 (0.4)	−4.5 (1.4)	−1.2 (0.5)		−1.4 (0.6)	−2.4 (0.4)	−1.8 (0.3)
Rel. humidity	1.7 (0.7)						−1.6 (0.3)
Abs. humidity							
Rainfall		4.8 (1.4)	2.1 (0.9)	1.1 (0.3)	4.5 (1.1)	−6.2 (2.0)	2.6 (0.9)
Solar radiation		5.1 (1.6)					
Wind velocity (EW)		3.4 (1.6)				−5.8 (1.3)	
Wind velocity (NS)						5.1 (1.5)	
AIC	262.5	247.0	259.0	251.6	262.7	216.6	208.7
Adj-R^2 (%)	66.5	36.9	48.8	34.6	47.2	84.1	67.2

[a] Estimates of meteorological variable were in 10^{-9} unit; Rel. humidity: Relative humidity; Abs. humidity: Absolute humidity; Wind velocity (EW): Wind velocity for East-to-West; Wind velocity (NS): Wind velocity for North-to-South; AIC: Akaike information criterion; Adj-R^2: Adjusted R-square in %.

Table S2. Pearson correlation coefficients and variance inflation factor between meteorological variables.

	Temperature	Rel. Humidity	Abs. Humidity [a]	Rainfall	Solar Radiation	Wind Velocity (EW)	Wind Velocity (NS)
Temperature		0.32	-	0.34	0.51	−0.01	0.00
Rel. humidity			-	0.45	−0.36	−0.10	0.03
Abs. humidity				0.46	0.34	−0.05	0.01
Rainfall					−0.20	−0.01	0.05
Solar radiation						0.01	0.00
Wind velocity (EW)							0.03
VIF	2.62	1.99	1.69	1.46	2.56	1.02	1.00

[a] Abs. humidity is derived from temperature and Rel. humidity so no correlation coefficients were drawn; Rel. humidity: Relative humidity; Abs. humidity: Absolute humidity; Wind velocity (EW): Wind velocity for East-to-West; Wind velocity (NS): Wind velocity for North-to-South; VIF: Variance inflation factor.

Table S3. Estimates (standard error) of significant meteorological determinants on changes of transmission rates for pooled data from 2002 to 2008.

Variables [a]	Estimate (SE)
Temperature	−1.2 (0.4)
Rel. humidity	2.5 (0.4)
Abs. humidity	
Rainfall	−2.1 (0.9)
Solar radiation	
Wind velocity (EW)	
Wind velocity (NS)	2.4 (0.8)
AIC	2034.3
Adj-R^2 (Meteorological)	24.0
Adj-R^2 (Meteorological + between season effect) [b]	43.9

[a] Estimates of meteorological variable were in 10^{-9} unit; [b] Adj-R^2 (Meteorological + between season effect): Adjusted R-square adjusting with the between-season effect by adding a component of year in the linear formula 4 of the main text; Rel. humidity: Relative humidity; Abs. humidity: Absolute humidity; Wind velocity (EW): Wind velocity for East-to-West; Wind velocity (NS): Wind velocity for North-to-South; SE: standard error; AIC: Akaike information criterion; Adj-R^2: Adjusted R-square in %.

Table S4. Estimates (standard error) of significant meteorological determinants on changes of transmission rates when fitting with P&I excess mortality *.

Variables [a]	Year						
	2002	2003 [b]	2004	2005	2006	2007	2008
Temperature	−1.4 (0.2)		−3.1 (1.1)		−2.1 (0.7)	−2.0 (0.5)	−3.1 (1.0)
Rel. humidity	2.5 (0.4)				1.8 (0.5)	−2.0 (0.7)	
Abs. humidity				3.1 (1.0)			
Rainfall				1.1 (0.3)			3.5 (1.7)
Solar radiation			3.5 (1.3)		1.6 (0.6)		7.1 (2.0)
Wind velocity (EW)			−3.3 (1.1)		−1.9 (0.5)	−9.8 (1.3)	2.8 (1.1)
Wind velocity (NS)	−2.0 (0.4)		3.8 (1.2)			2.0 (0.8)	
AIC	98.9	-	145.7		198.9	172.7	188.9
Adj-R^2 (%)	71.9	-	38.9		51.5	84.7	48.0

* The excess P&I mortality was estimated as the difference between the observed the upper 95% limit of the prediction interval of baseline deaths. The seasonal baseline was obtained by using a Serfling regression model: $E(Y_t) = \beta_0 + \beta_1 t + \beta_2 t^2 + \beta_3 t^3 + \beta_4 \sin(2\pi t/52.17) + \beta_5 \cos(2\pi t/52.17)$ where $E(Y_t)$ represents the expected baseline weekly number of P&I deaths at week t. The β_0 is the intercept. The β_1, β_2 and β_3 accounts for the linear, quadratic and cubic terms of time trends respectively. The functions sin and cos are harmonic terms representing annual periodicity. [a] Estimates of meteorological variable were in 10^{-7} unit; [b] No parameters showed significant effect; Rel. humidity: Relative humidity; Abs. humidity: Absolute humidity; Wind velocity (EW): Wind velocity for East-to-West; Wind velocity (NS): Wind velocity for North-to-South; AIC: Akaike information criterion; Adj-R^2: Adjusted R-square in %.

© 2015 by the authors; licensee MDPI, Basel, Switzerland. This article is an open access article distributed under the terms and conditions of the Creative Commons Attribution license (http://creativecommons.org/licenses/by/4.0/).
